# Bearing Fault Diagnosis by a Robust Higher-Order Super-Twisting Sliding Mode Observer

**DOI:** 10.3390/s18041128

**Published:** 2018-04-07

**Authors:** Farzin Piltan, Jong-Myon Kim

**Affiliations:** 1 Department of Electrical, Electronics and Computer Engineering, University of Ulsan, Ulsan 680-479, Korea; piltan_f@iranssp.org; 2 School of IT Convergence, University of Ulsan, Ulsan 680-479, Korea

**Keywords:** Model-reference fault diagnosis, bearing fault diagnosis, super-twisting higher-order sliding mode observation technique, ARX-Laguerre proportional integral observation method

## Abstract

An effective bearing fault detection and diagnosis (FDD) model is important for ensuring the normal and safe operation of machines. This paper presents a reliable model-reference observer technique for FDD based on modeling of a bearing’s vibration data by analyzing the dynamic properties of the bearing and a higher-order super-twisting sliding mode observation (HOSTSMO) technique for making diagnostic decisions using these data models. The HOSTSMO technique can adaptively improve the performance of estimating nonlinear failures in rolling element bearings (REBs) over a linear approach by modeling 5 degrees of freedom under normal and faulty conditions. The effectiveness of the proposed technique is evaluated using a vibration dataset provided by Case Western Reserve University, which consists of vibration acceleration signals recorded for REBs with inner, outer, ball, and no faults, i.e., normal. Experimental results indicate that the proposed technique outperforms the ARX-Laguerre proportional integral observation (ALPIO) technique, yielding 18.82%, 16.825%, and 17.44% performance improvements for three levels of crack severity of 0.007, 0.014, and 0.021 inches, respectively.

## 1. Introduction

Rolling element bearings (REBs) are very important components in rotating machines, as they are used to reduce the friction between moving parts for linear and rotational motion [1]. Bearings have been widely used in the rotating machinery in various industries, such as steel mills, paper mills, and wind power generators, to improve their lifespan and efficiency by reducing friction and facilitating motion [2]. Complexities of the tasks and nonlinear parameters in REBs make their fault detection and diagnosis (FDD) very challenging. The detection and diagnosis (FDD) of faults is necessary to prevent the complete failure of the bearing and hence avoid the impairment of the machinery. Several types of faults have been defined in REBs, which are divided into four main categories, i.e., inner raceway faults, outer raceway faults, ball faults, and cage faults [3].

Different techniques have been introduced for the diagnosis of faults in bearings, including signal-based fault diagnosis [4,5,6,7,8,9], knowledge-based fault diagnosis [10,11], model-based fault diagnosis [12,13,14], and hybrid/active approaches to fault diagnosis [15,16]. Although signal-based fault diagnosis has several advantages, this method has challenges associated with system reliability in the presence of uncertainty and external disturbances. Knowledge-based fault diagnosis has its own challenges, as it requires massive quantities of data for training the system to make diagnostic decisions. Model-based fault diagnosis identifies the faults by using a small dataset, but it needs to model the system’s dynamics [17]. Various model-based methods have been rigorously studied in the field of detection, isolation, and identification for REBs [1,17]. Model-reference methodologies detect faults by setting a threshold for the residual signal, which is generated from the difference between an actual signal and the system’s estimation of that signal [18]. These residuals are highly sensitive to the possible faults in the system, which can affect the diagnostic performance [2,19]. These signals are certainly independent of the inputs and outputs under normal conditions. Model-reference based fault diagnosis utilizes output observers, system identification and parameter estimation, and the parity equation [1,12,20,21].

Specifically, the system-observer-based technique is regarded as an important model-reference methodology for FDD [1]. Observation methods are designed using different algorithms, such as the proportional-integral (PI) observation technique [22,23], the proportional multiple-integral (PMI) observation method [24,25,26], the descriptor observation technique [27,28], adaptive observation methods [29,30,31], and sliding mode observation techniques [14,32,33,34,35]. Sliding mode observer (SMO) is an excellent FDD candidate for systems that operate in uncertain and noisy conditions. In this technique, the output estimation error is forced to zero based on the nonlinear switching term. This method can detect and isolate a fault as it adaptively updates the system parameters, which can significantly improve the diagnostic performance of this method if applied for FDD in bearings. Furthermore, this observer works based on the system’s behavior, which tends to work very well when most of the dynamic and physical parameters are adequately known [36,37,38,39]. Apart from several advantages, such as stability and reliability, this method of using an SMO suffers from the chattering phenomenon, and requires the relative degree of the outputs concerning the uncertainties or disturbances to be one. In mechanical systems based on the position observation, the estimation of the first and second derivative of position, such as velocity and acceleration, respectively, is necessary. Thus, in the acceleration equation, uncertainties and external disturbances are relative to the second derivative of the measured position [39]. The higher-order sliding mode observer (HOSMO) has been proposed to improve the performance of SMO in the presence of uncertainty and disturbances [40,41,42,43,44,45]. Since HOSMO employs a discontinuous control algorithm on the higher-order derivatives, chattering can be attenuated by moving the switching to the higher derivatives in HOSMO. The performance of the higher-order sliding mode technique has been improved by using different algorithms, such as the suboptimal algorithm [46], the quasi-continuous technique [47], and the twisting method [48]. Apart from the many advantages of sub-optimal HOSMC, the quasi-continuous HOSMC, and the twisting HOSMC, these methods face a critical challenge related to the first-order derivative of the sliding variable. This issue has been addressed by proposing a higher-order super-twisting sliding mode technique [38]. For unmeasurable state observers and high-accuracy velocity estimation without filtration, the higher-order super-twisting sliding mode observer (HOSTSMO) was proposed [32,33,49,50]. In this paper, we propose a robust higher-order super-twisting sliding mode observer for fault detection and isolation in the presence of uncertainty and external disturbances for rolling element bearings (REBs).

The rest of this paper is organized as follows. Section 2 gives the problem statements and fault diagnosis objectives. Section 3 presents the detailed mathematical modeling of an REB with 5 degrees of freedom. Section 4 shows a comprehensive methodology to design a robust higher-order super-twisting sliding mode observation technique for fault detection and diagnosis. Datasets, results, and discussion are presented in Section 5. Section 6 concludes this paper.

## 2. Problem Statements and Fault Diagnosis Objectives

**Figure 1:**
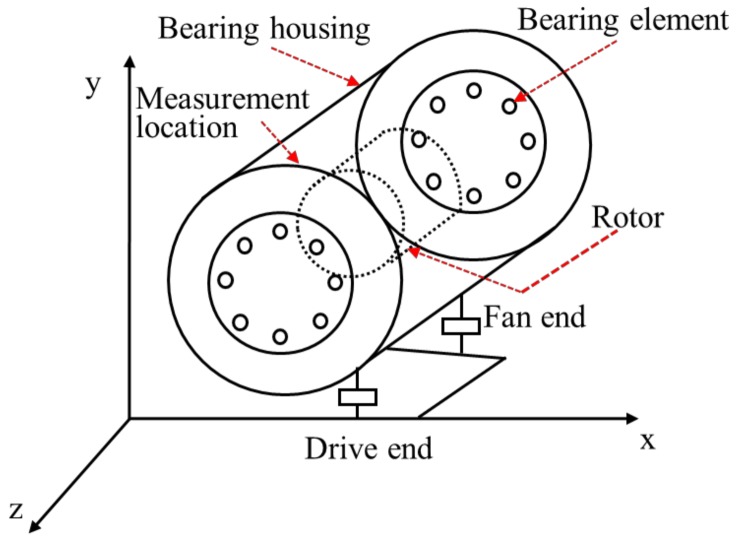
The system geometry, measurement location, and configuration of the system.

The main objective of this paper is to devise a robust scheme for the detecting and estimating of faults in rolling element bearings (REBs), including inner, outer, and roller faults. The proposed scheme is based on vibration modeling and a higher-order sliding mode observer in the presence of uncertainty and disturbance. The foremost challenge is to model the REB vibration data in terms of the energy. This paper utilizes vibration data collected using an experimental testbed, which is illustrated in Figure 1 [2]. The corresponding Lagrangian formulation for this system consists of potential energy, kinetic energy, and generalized forces as shown below:

(1) $$\frac{d}{dt}\left(\frac{\partial K}{\partial {\dot{q}}_{i}}\right)-\frac{\partial K}{\partial q_{i}}+\frac{\partial P}{\partial q_{i}}=Q_{i},\;i=1,2,3,\;\ldots ,n_{DOF},$$

where *K* is the kinetic energy, *P* represents the potential energy, $Q_{i}$ represents a generalized force, $q_{i}$ is the generalized coordinate, and $n_{DOF}$ is the number of degrees of freedom. Each generalized coordinate corresponds to a degree of freedom (DOF) of the system, and each generalized force in the system acts along the corresponding generalized coordinate. The energy equation is obtained by taking the derivative of Equation (1) with respect to each generalized coordinate as follows:

(2) $$F_{\left(\theta \right)}=M_{\left(\theta \right)}[\ddot{\theta }]+H(\theta ,\dot{\theta })+\varphi +\Delta +\delta (t-T_{f}),$$

where $F_{\left(\theta \right)},M_{\left(\theta \right)},\varphi ,\Delta ,\delta ,(t-T_{f})$ and $T_{f}$ are the force vector, time-variant mass matrix, time-variant nonlinear bearing vector, unknown modeling parameters, faults vector (inner, outer, and ball), time profile of the faults, and time of fault occurrence, respectively. If $H(\theta ,\dot{\theta })=C(\theta )[\dot{\theta }]+K(\theta )[\theta ]$ and $\Delta =(\Delta M)(\theta )[\ddot{\theta }]+(\Delta C)(\theta )[\dot{\theta }]+(\Delta K)(\theta )[\theta ]$, then the Lagrange dynamic formulation of a bearing can be written as follows:

(3) $$F_{\left(\theta \right)}=(M+\Delta M)(\theta )[\ddot{\theta }]+(C+\Delta C)(\theta )[\dot{\theta }]+(K+\Delta K)(\theta )[\theta ]+\varphi +\delta (t-T_{f}),$$

where C(θ),K(θ) and (ΔM,ΔC,ΔK) are the time-variant stiffness matrix, time-variant damping matrix, and unknown modeling parameters for mass, stiffness, and damping matrices, respectively.

To simplify the modeling and analysis, (2) and (3) are re-written as follows:

(4) $$[\ddot{\theta }]=[M^{-1}(\theta )]\times (F_{\left(\theta \right)}-\Psi (\theta ,\dot{\theta }))-\lambda (\theta ,\dot{\theta },t),$$

where $\Psi (\theta ,\dot{\theta })=C(\theta )[\dot{\theta }]+K(\theta )[\theta ]+\varphi$ and $\lambda (\theta ,\dot{\theta },t)={M^{-1}}_{\left(\theta \right)}\times (\Delta +\delta (t-T_{f}))$ represent the modeling uncertainty and faults of the bearings. For a bearing in healthy condition, it is assumed that the uncertainty is bounded as follows:

(5) $$if(t<T_{f})\to \delta (t-T_{f})=0\to \left\|M^{-1}(\theta )\times \Delta \right\|\leq \Gamma ,$$

where Γ is a constant. In the faulty condition, (5) can be written as follows:

(6) $$if(t>T_{f})\to \left\|{M^{-1}}_{\left(\theta \right)}\times (\Delta +\delta (t-T_{f}))\right\|=\lambda (\theta ,\dot{\theta },t)>\Gamma .$$

**Figure 2:**
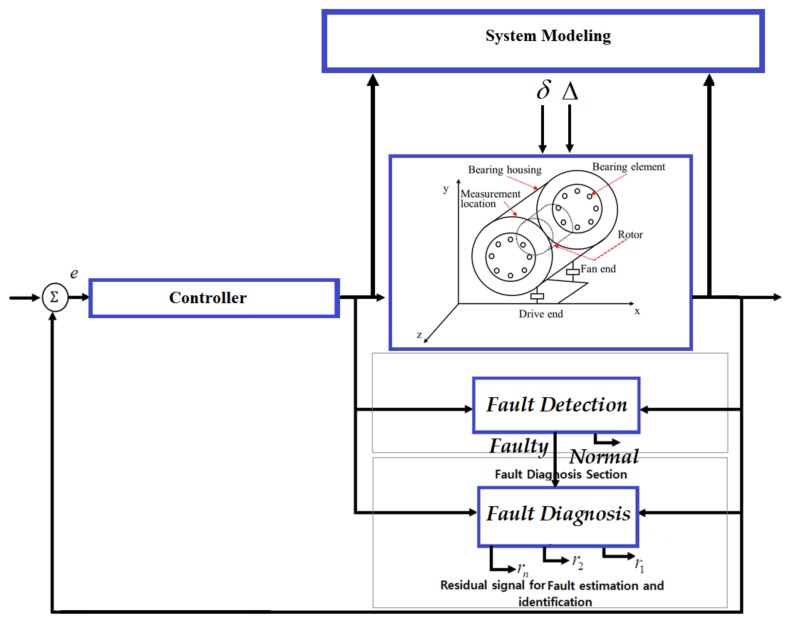
A block diagram of systems, faults, and fault diagnosis and their associations.

Based on the above formulations, we can see that mathematical modeling of REBs is very complicated, and it is not exact. Moreover, the model’s behavior may be different from the real system’s behavior in both healthy and faulty conditions because the model is usually obtained under various assumptions that may not hold true for a real system. This makes the detection and diagnosis of faults in rolling element bearings more challenging and warrants the development of an algorithm that is robust to modeling uncertainties and disturbances. To solve the challenge of uncertain parameters in system modeling, a higher-order super-twisting sliding mode observer is recommended in this study. This observation technique estimates the faults based on robust model-based nonlinear methods and improves the rate of fault detection and diagnosis. The objectives of fault diagnosis for an REB in the presence of uncertainty is the estimation of inner, outer, and ball faults based on model reference HOSTSMO, which is defined as follows:

(7) $$\begin{array}{l} \left[{\delta_{i}}_{estimate}]\to [{\delta_{i}}_{d}\right]\\ {}[{\delta_{o}}_{estimate}]\to [{\delta_{o}}_{d}],\\ \left[{\delta_{b}}_{estimate}]\to [{\delta_{b}}_{d}\right] \end{array}$$

where $\left[{\delta_{i}}_{estimate}],[{\delta_{i}}_{d}],[{\delta_{o}}_{estimate}],[{\delta_{o}}_{d}],[{\delta_{b}}_{estimate}\right]$ and $\left[{\delta_{b}}_{d}\right]$ are the estimated inner fault, desired inner fault, estimated outer fault, desired outer fault, estimated ball fault, and desired ball fault, respectively. The block diagram of systems, faults, and fault diagnosis in an REB is illustrated in Figure 2.

## 3. Mathematical Modeling of REBs

As bearing data is inherently nonlinear, we choose the HOSTSMO technique for fault detection and diagnosis. This robust method is highly efficient and can provide excellent detection and diagnostic performance. The HOSTSMO technique offers a flexible way to find the optimized parameters for a nonlinear data model.

**Figure 3:**
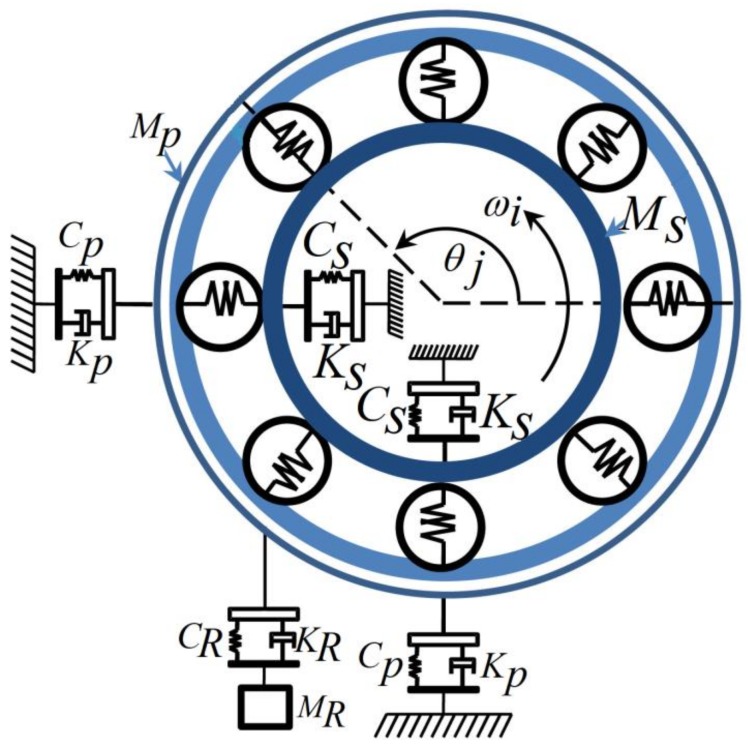
Five degrees of freedom of the rolling element bearing (REB) [53].

The mathematical model of the REB can be expressed in terms of the angular position of the ball, the fundamental train frequency (FTF), and time, using the following formulations [51,52,53]:

(8) $$\begin{array}{l} \theta_{j}=\frac{2\Pi (j-1)}{n_{b}}+\omega_{c}t+\theta_{0}\\ \omega_{c}=\frac{\omega_{i}}{2}(1-\frac{d}{D}) \end{array},$$

where *θ_j_*, *n_b_*, *ω_c_*, ***ω_i_***, *t*, *θ*_0_, *d*, and *D* are the angular position of the *j*-th ball, number of balls, FTF, constant rotor velocity, elapsed time, initial position, ball diameter, and pitch diameter of the bearing, respectively. Figure 3 illustrates the 5 degrees of freedom for modeling the REB.

The contact forces are defined by the following equations [52,53]:

(9) $$\begin{array}{l} F_{x}=\sum_{j=1}^{N_{b}}C_{p}\delta_{j}^{\gamma }\cos(\theta_{j}).h(-\delta_{j})\\ F_{y}=\sum_{j=1}^{N_{b}}C_{p}\delta_{j}^{\gamma }\cos(\theta_{j}).h(-\delta_{j}) \end{array}$$

(10) $$h(x)=\{\begin{array}{cc} 1 & x\geq 0\\ 0 & x<0 \end{array}.$$

The contact deformation is defined as follows:

(11) $$\delta_{j}={\theta_{x}}_{d}.Cos(\theta_{j})+{\theta_{y}}_{d}.Sin(\theta_{j}-\omega ).$$

Here, $\delta_{j},\theta_{j},\gamma ,h(x),C_{p},\theta_{xd}$, and ${\theta_{y}}_{d}$ are the contact deformation, angular position of the *j*-th REB, force exponent, Heaviside function, stiffness of outer race, and displacements between inner race and ball in the inner, outer, and ball faults in the *x* and *y* directions, respectively. Based on [51,53], the 5-DOF REB model has three main parts: an outer race, which is modeled by 2-DOF, an inner race, which is modeled by 2-DOF, and the sprung mass, which is modeled by 1-DOF. The equation of the outer race is defined as follows [53]:

(12) $$\begin{array}{l} M_{p}{{\ddot{\theta }}_{x}}_{o}=F_{x}-K_{p}{{\dot{\theta }}_{x}}_{o}-C_{p}{\theta_{x}}_{o}\\ M_{p}{{\ddot{\theta }}_{y}}_{o}=F_{y}-M_{p}g-(K_{p}+K_{R}){{\dot{\theta }}_{y}}_{o}-(C_{p}+C_{R})\theta y_{o}+C_{R}{\theta_{y}}_{R}+K_{R}{{\dot{\theta }}_{y}}_{R} \end{array},$$

where $M_{p},{\theta_{x}}_{o},{\theta_{y}}_{o},K_{p},g,K_{R},C_{R}$, and ${\theta_{y}}_{R}$ are the outer mass, outer center of mass along the *x-axis*, outer center of mass along the *y-axis*, outer damping, gravity, damping of the sprung-mass, stiffness of the sprung-mass, and sprung-mass displacement, respectively. To model the inner race, the equation of the inner race is defined as follows [53]:

(13) $$\begin{array}{l} M_{s}{{\ddot{\theta }}_{x}}_{i}=-F_{x}+K_{s}{{\dot{\theta }}_{x}}_{i}+C_{s}{\theta_{x}}_{i}\\ M_{s}{{\ddot{\theta }}_{y}}_{i}=-F_{y}-M_{s}g-K_{s}{{\dot{\theta }}_{y}}_{i}-C_{s}{\theta_{y}}_{i} \end{array},$$

where $M_{s},{\theta_{x}}_{i},{\theta_{y}}_{i},K_{s}$, and $C_{s}$ are the mass of the shaft, inner center of mass along the *x-axis*, inner center of mass along the *y-axis*, damping of the shaft, and stiffness of the shaft, respectively. The sprung mass equation (1-DOF) along the *y-axis* is given as follows [53]:

(14) $$M_{R}{{\ddot{\theta }}_{y}}_{R}=C_{R}({\theta_{y}}_{o}-{\theta_{y}}_{R})+K_{R}({{\dot{\theta }}_{y}}_{o}-{{\dot{\theta }}_{y}}_{R})-m_{R}.g.$$

Here, $M_{R}$ is the mass of the sprung-mass. Based on [51,53], the localized faults for the outer race, inner race, and ball are given in the following equations. If centers of mass in the *x* and *y* directions are different, then the fault deformation is given by Equations (15) and (16):

(15) $$\begin{array}{l} {\theta_{x}}_{d}={\theta_{x}}_{i}-{\theta_{x}}_{o}\\ {\theta_{y}}_{d}={\theta_{y}}_{i}-{\theta_{y}}_{o} \end{array}\mathrm{and}$$

(16) $$\delta_{f}=\{\begin{array}{cc} \omega_{d} & \phi_{d}<\theta_{j}<\phi_{d}+\Delta \phi_{d}\\ 0 & otherwise \end{array},$$

whereas the outer contact deformation fault is defined as follows:

(17) $$\delta_{o}=\max({\theta_{x}}_{d}.Cos(\theta_{j})+{\theta_{y}}_{d}.Sin(\theta_{j})-\omega -\delta_{f},0).$$

The inner contact deformation fault is defined by Equation (20) if the specified angular position $\left(\phi_{d}\right)$ and fault deformation $\left(\delta_{f}\right)$ are given by Equations (18) and (19), respectively:

(18) $$\phi_{d}=\omega_{i}t+\phi_{0},$$

(19) $$\delta_{f}=\{\begin{array}{cc} \omega_{d} & \phi_{d}<\theta_{j}<\phi_{d}+\Delta \phi_{d}\\ 0 & otherwise \end{array},\;\mathrm{and}$$

(20) $$\delta_{i}=\max({\theta_{x}}_{d}.Cos(\theta_{j})+{\theta_{y}}_{d}.Sin(\theta_{j})-\omega -\delta_{f},0),$$

where $\left(\phi_{0}\right)$ and $\left(\delta_{i}\right)$ are the initial spall location and the inner contact deformation fault, respectively. If the fault deformation is expressed as follows:

(21) $$\delta_{f}=\{\begin{array}{cc} \omega_{dr}-\omega_{do} & 0<\phi_{s}<\phi_{bo}\\ \omega_{dr}+\omega_{do} & \Pi <\phi_{s}<\Pi +\phi_{bi}\\ 0 & otherwise \end{array},$$

where $\left(\phi_{b_{i}}\right)$ and $\left(\phi_{bo}\right)$ are the angular widths of inner and outer faults, respectively, then $\omega_{dr}$ and $\omega_{do}$ can be given as follows:

(22) $$\omega_{dr}=\frac{1}{2[d-\sqrt{(d^{2}-4x^{2}})]},$$

(23) $$\omega_{do}=\frac{1}{2[D_{o}-\sqrt{({D_{o}}^{2}-4x^{2}})]}.$$

Therefore, the ball contact deformation fault is then defined as follows:

(24) $$\delta_{b}=\max({\theta_{x}}_{d}.\mathrm{Cos}(\theta_{j})+{\theta_{y}}_{d}.\mathrm{Sin}(\theta_{j})-\omega -\delta_{f},0).$$

Thus, the rolling element bearing model in the presence of uncertainty and faults can be expressed as follows:

(25) $$F_{\left(\theta_{x,y}\right)}=[M_{\left(\theta \right)}]\ddot{\theta }+[C_{\left(\theta \right)}]\dot{\theta }+[K_{\left(\theta \right)}]\theta +\varphi +F_{d(\dot{\theta })}+(\delta_{i}(t-T_{f_{i}}))+(\delta_{o}(t-T_{f_{o}}))+(\delta_{b}(t-T_{f_{b}})),$$

**Figure 4:**
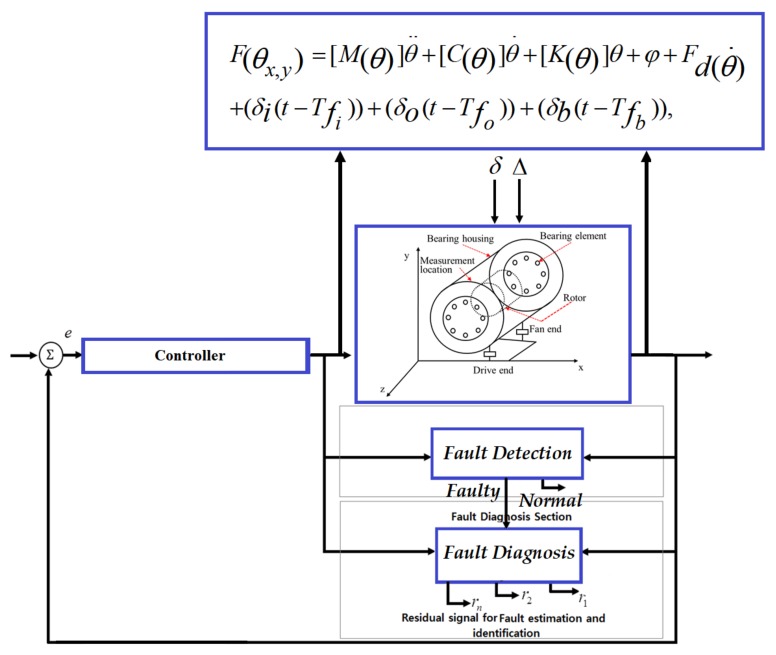
Mathematical modeling of bearing.

The block diagram of the mathematical model of an REB is illustrated in Figure 4. To design a model-reference-based fault diagnosis scheme for bearings, this paper uses a 5-DOF mathematical model for an REB system and a benchmark bearing dataset, which was acquired from Case Western Reserve University (CWRU) [54]. The data is collected using vibration acceleration sensors installed on the bearing housings. The bearings used for the collection of this data are 6205-2RS JEM SKF roller bearings, and their parameters for the 5 degrees of freedom model are given in Table 1 [55,56].

## 4. Proposed Method

The vibration signals of an REB have various types of disturbances. Thus, designing a robust approach for fault detection and diagnosis is the principal challenge. In the first step, ARX-Laguerre proportional integral observer (APIO) is briefly discussed. The primary challenge of this technique is robustness. To address this challenge, the proposed higher-order super-twisting sliding mode observer (HOSTSMO) is the second candidate for fault diagnosis in an REB. This technique is designed to ensure fast convergence of the estimated faults to the measured faults in the presence of uncertainties, and to attenuate the chattering.

### 4.1. ARX-Laguerre Proportional-Integral Observer (APIO) 

As the rolling element bearing is a nonlinear system, if.. and $X_{2}=\dot{\theta }$, the state-space formulation for an REB can be given as follows:

(26) $$\left\{ \begin{array}{l} {\dot{X}}_{1}=X_{2}=\dot{\theta },\\ {\dot{X}}_{2}=\alpha (X_{1},X_{2},u)+\Delta (X_{1},X_{2},t)\\ Y={\left(K\right)}^{T}X_{1}, \end{array}\right.+\delta_{i}(t)+\delta_{o}(t)+\delta_{b}(t),$$

where u=F(θ), $\alpha (X_{1},X_{2},u)=M^{-1}(\theta )\times (F(\theta )-\Psi (\theta ,\dot{\theta }))$, $\left({\dot{X}}_{1},{\dot{X}}_{2}\right)$ are system states, K is a coefficient, u is the control input, $\delta_{i}(t)$ is the inner fault, $\delta_{o}(t)$ is the outer fault, $\delta_{b}(t)$ is the ball fault, $\Delta (X_{1},X_{2},t)$ is the system uncertainty, and Y is the measured output. The ARX-Laguerre orthonormal technique is given as follows [57]:

(27) $$\begin{array}{l} Y(K)=\sum_{0}^{N_{a}-1}K_{n,a}(\sum_{j=1}^{\infty }\frac{\sqrt{1-{\zeta_{a}}^{2}}}{Z-\zeta_{a}}{\left(\frac{1-\zeta_{a},z}{Z-\zeta_{a}}\right)}^{n}*y(k)).x_{n,y}(k)+\\ \sum_{0}^{N_{b}-1}K_{n,b}(\sum_{j=1}^{\infty }\frac{\sqrt{1-{\zeta_{b}}^{2}}}{Z-\zeta_{b}}{\left(\frac{1-\zeta_{b},z}{Z-\zeta_{b}}\right)}^{n}*u(k)).x_{n,u}(k) \end{array}$$

where $Y(K),u(k),(K_{n,a}\;\&\;K_{n,b}),(N_{a},N_{b}),(\frac{\sqrt{1-{\zeta_{i}}^{2}}}{Z-\zeta_{i}}{\frac{1-{\zeta_{i}}^{2},z}{Z-\zeta_{i}}}^{n}),\xi_{i},*,x_{n,y}(k)$, and $x_{n,u}(k)$ are the system output, system input, Fourier coefficients, system order, Laguerre-based orthonormal function, Laguerre pole, convolution product, output signal filter, and input signal filter, respectively. The state space equation for the ARX-Laguerre orthonormal function can be written as follows:

(28) $$\left\{ \begin{array}{l} X(k)=[AX(k-1)+b_{y}y(k-1)+b_{u}u(k-1)]+\Delta (k-1)+\delta (k-1)]\\ Y(k)={\left(K\right)}^{T}X(k) \end{array}\right.$$

where $X(k),Y(k),u(k),\Delta (k),\delta (k),(A,b_{y},b_{u})$ and ${\left(K\right)}^{T}$ are the input/output filter, measured output, control input, uncertainty and disturbance, faults, coefficient matrices, and the Fourier coefficient, respectively. The matrix A is given as follows:

(29) $$A=\left[\begin{array}{cc} A_{y} & O_{N_{a},N_{b}}\\ O_{N_{b},N_{a}} & A_{u} \end{array}\right],$$

(30) $$\left\{ \begin{array}{l} A_{y}=\left[\begin{array}{cccc} \zeta_{a} & 0 & \ldots & 0\\ 1-{\zeta_{a}}^{2} & \zeta_{a} & \ldots & 0\\ -\zeta_{a}(1-{\zeta_{a}}^{2}) & 1-{\zeta_{a}}^{2} & \ldots & 0\\ \begin{array}{c} \ldots \\ \ldots \\ {\left(-\zeta_{a}\right)}^{N_{a}-1}(1-{\zeta_{a}}^{2}) \end{array} & \begin{array}{c} \ldots \\ \ldots \\ \ldots \end{array} & \begin{array}{c} \ldots \\ \ldots \\ \ldots \end{array} & \begin{array}{c} 0\\ 0\\ \zeta_{a} \end{array} \end{array}\right]\\ A_{u}=\left[\begin{array}{cccc} \zeta_{b} & 0 & \ldots & 0\\ 1-\zeta_{b}{}^{2} & \zeta_{a} & \ldots & 0\\ -\zeta_{b}(1-{\zeta_{b}}^{2}) & 1-{\zeta_{b}}^{2} & \ldots & 0\\ \begin{array}{c} \ldots \\ \ldots \\ {\left(-\zeta_{b}\right)}^{N_{b}-1}(1-{\zeta_{b}}^{2}) \end{array} & \begin{array}{c} \ldots \\ \ldots \\ \ldots \end{array} & \begin{array}{c} \ldots \\ \ldots \\ \ldots \end{array} & \begin{array}{c} 0\\ 0\\ \zeta_{b} \end{array} \end{array}\right] \end{array}\right.,,$$

$O_{N_{a},N_{b}}$ and $O_{N_{b},N_{a}}$ are null matrices of dimensions $N_{a}\times N_{b}$ and $N_{b}\times N_{a}$, respectively. The vectors $b_{y}$ and $b_{u}$ can be defined as follows:

(31) $$b_{y}=\sqrt{1-\zeta_{a}^{2}}\left[\begin{array}{c} 1\\ -\zeta_{a}\\ {\left(-\zeta_{a}\right)}^{2}\\ \begin{array}{c} ..\\ ..\\ {\left(-\zeta_{a}\right)}^{N_{a}-1} \end{array} \end{array}\right]$$

(32) $$b_{u}=\sqrt{1-\zeta_{b}^{2}}\left[\begin{array}{c} 1\\ -\zeta_{b}\\ {\left(-\zeta_{b}\right)}^{2}\\ \begin{array}{c} ..\\ ..\\ {\left(-\zeta_{b}\right)}^{N_{b}-1} \end{array} \end{array}\right].$$

The ARX-Laguerre PI observer for a faulty system is given as follows:

(33) $$\left\{ \begin{array}{l} \hat{X}(k)=[A\hat{X}(k-1)+b_{y}\hat{Y}(k-1)+b_{u}u(k-1)]+\hat{\Delta }(k-1)\\ +{\hat{\delta }}_{i}(k-1)+{\hat{\delta }}_{o}(k-1)+{\hat{\delta }}_{b}(k-1)+K_{p}[Y(k-1)-\hat{Y}(k-1)]]\\ \hat{Y}(k)={\left(K_{\alpha }\right)}^{T}\hat{X}(k)\\ {\hat{\delta }}_{i}(k)={\hat{\delta }}_{i}(k-1)+K_{ii}[Y(k-1)-\hat{Y}(k-1)]\\ {\hat{\delta }}_{o}(k)={\hat{\delta }}_{o}(k-1)+K_{io}[Y(k-1)-\hat{Y}(k-1)]\\ {\hat{\delta }}_{b}(k)={\hat{\delta }}_{b}(k-1)+K_{ib}[Y(k-1)-\hat{Y}(k-1)] \end{array}\right.$$

where $\hat{X}(k),\hat{Y}(k),u(k),\hat{\Delta }(k),{\hat{\delta }}_{i}(k),{\hat{\delta }}_{o}(k),{\hat{\delta }}_{b}(k)$ and $\left(K_{\alpha },K_{p},K_{ii},K_{io},K_{ib}\right)$ are the estimated system state for (inner, outer, and ball) faulty conditions, estimated measured output for (inner, outer, and ball) faulty conditions, control input, estimated uncertainty and disturbance, estimated inner fault, estimated outer fault and estimated ball fault, and gains, respectively. Gains are optimized based on the Linear Matrix Inequality (LMI) optimization method as follows:

(34) $$\left\{ \begin{array}{l} (1-2\gamma )P-(A_{\gamma }^{T}P-K_{\gamma }^{T})P^{-1}(PA_{\gamma }-K_{\gamma })>0\\ \gamma \in [0,0.5]\\ K_{\varepsilon }=\left[\begin{array}{c} K_{P}\\ K_{i*} \end{array}\right],*=i,o,b\\ K_{\gamma }=\left[\begin{array}{cc} K^{T} & 0 \end{array}\right]\\ A_{\gamma }=\left[\begin{array}{cc} A+b_{y}C^{T} & b_{u}\\ 0_{1,M} & 1 \end{array}\right] \end{array}\right.$$

where γ is the decay rate that is used to quantify the convergence rate of the estimation error and P is the Lyapunov symmetric and positive definite matrix. The ALPIO is able to detect the system faults. According to (5), $\delta (t-T_{f})=0$ when $t<T_{f}$, the system works in a healthy condition and the residual is defined as follows:

(35) $$r(k)=Y(k)-\hat{Y}(k)\leq \Gamma .$$

Based on (6), in faulty conditions, $\delta (t-T_{f})\neq 0$ when $t>T_{f}$, then the residual signal is defined by

(36) $$r(k)=Y(k)-\hat{Y}(k)>\Gamma .$$

Since in normal condition the residual r(k) is smaller than Γ, whereas in a faulty state it is greater than Γ, Γ is therefore defined as the threshold value for fault detection. Based on (33), the ball, inner, and outer faults can be estimated using the ALPIO method. To calculate the threshold value for the ball, inner, and outer fault conditions, we can define an error control term for the error compensator for the ball, inner, and outer thresholds as follows:

(37) $$\Delta_{b}=-M(\hat{X}).(K_{p}r_{b}+{\dot{r}}_{b}).$$

(38) $$\Delta_{i}=-M(\hat{X}).(K_{p}r_{i}+{\dot{r}}_{i}).$$

(39) $$\Delta_{o}=-M(\hat{X}).(K_{p}r_{i}+{\dot{r}}_{i})$$

where $\Delta_{i},\Delta_{b}$ and $\Delta_{o}$ are the threshold values for inner fault, ball fault, and outer fault, respectively. Based on (37)–(39), the ball, inner, and outer faults can be identified as follows:

(40) $$\begin{array}{l} r=[Y]-[\hat{Y}]\\ if:r>\Gamma ,r<\Delta_{b},r<\Delta_{i},r<\Delta_{o}\to r=r_{b}\\ if:r>\Gamma ,r>\Delta_{b},r<\Delta_{i},r<\Delta_{o}\to r=r_{i}\\ if:r>\Gamma ,r>\Delta_{b},r>\Delta_{i},r<\Delta_{o}\to r=r_{o} \end{array}$$

where $r_{b},r_{i}$ and are the residual signals for ball fault, inner fault, and outer fault, respectively. Based on (40), the fault can be isolated whenever the residuals $\left(r_{b},r_{i},r_{o}\right)$ overshoot their corresponding thresholds $\left(\Gamma ,\Delta_{b},\Delta_{i}\right)$, respectively. Though useful in many cases, this method is not robust in detecting and isolating faults in the presence of uncertainties and disturbances. To improve its robustness, a sliding mode observer is used.

### 4.2. Proposed Higher-Order Super-Twisting Sliding Mode Observer (HOSTSMO)

The simple sliding mode observer is defined as follows [39]:

(41) $$\left\{ \begin{array}{l} \begin{array}{cc} {\dot{\hat{X}}}_{1}={\hat{X}}_{2}+\lambda_{a}.\mathrm{sgn}(e_{1}), & \left(e_{1}=X_{1}-{\hat{X}}_{1}\right) \end{array}\\ \begin{array}{cc} {\dot{\hat{X}}}_{2}=\alpha (X_{1},{\hat{X}}_{2},u)+\lambda_{b}.\mathrm{sgn}(e_{2}), & \left(e_{2}={\dot{\hat{X}}}_{1}-{\hat{X}}_{2}\right) \end{array}\\ \hat{Y}={\left(K_{\beta }\right)}^{T}{\hat{X}}_{1} \end{array}\right.$$

where u=F(θ), $\alpha (X_{1},X_{2},u)=M^{-1}(\theta )\times (F(\theta )-\Psi (\theta ,\dot{\theta }))$, $\left({\hat{\dot{X}}}_{1},{\hat{\dot{X}}}_{2}\right)$ are estimated system states, $\left(K_{\beta },\lambda_{a},\lambda_{b}\right)$ are coefficients, u is the control input, and $\hat{Y}$ is the estimated measured output. The SMO is stable and robust; however, it suffers from the chattering phenomenon. The new part is defined as follows:

(42) $$H=\begin{array}{cc} \lambda {\left|e_{i}\right|}^{0.5}\mathrm{sgn}(e_{i}) & ,\lambda >0, \end{array}$$

where H and λ are an observation function and coefficient, respectively. If the uncertainties are estimated, the sliding dynamics can converge to zero in finite time.

(43) $$\left\{ \begin{array}{l} H=\lambda {\left\|e_{i}\right\|}^{0.5}\mathrm{sgn}(e_{i})-\hat{\chi }\\ \hat{\dot{\chi }}=-\lambda_{0}\times \mathrm{sgn}(e_{i}) \end{array}\right.,$$

where $\hat{\dot{\chi }}$ and $\lambda_{0}$ are the super-twisting variable and coefficient, respectively. The compensate sliding variable dynamic is defined as follows:

(44) $$\left\{ \begin{array}{l} \lambda {\left\|e_{i}\right\|}^{0.5}\mathrm{sgn}(e_{i})-\hat{\chi }=\hat{\lambda }(X_{1},X_{2},t)\\ \hat{\dot{\chi }}=-\lambda_{0}\times \mathrm{sgn}(e_{i}) \end{array}\right.$$

where $\lambda (\theta ,\dot{\theta },t)={M^{-1}}_{\left(\theta \right)}\times (\Delta +\delta (t-T_{f}))$ represents the modeling uncertainty and estimated bearing faults. Based on (43) and (44), the challenge of uncertainties and unknown inputs (faults) estimation can be solved in finite time. Equation (44) is called the super-twisting algorithm. Based on (43), the formulation of HOSTSMO can be given as follows:

(45) $$\left\{ \begin{array}{l} \begin{array}{cc} {\dot{\hat{X}}}_{1}={\hat{X}}_{2}+\lambda_{1}{\left|e_{1}\right|}^{2/3}.\mathrm{sgn}(e_{1}), & \left(e_{1}=X_{1}-{\hat{X}}_{1}\right) \end{array}\\ \begin{array}{cc} {\dot{\hat{X}}}_{2}=\alpha (x_{1},{\hat{x}}_{2},u)+\lambda_{2}{\left|e_{2}\right|}^{0.5}.\mathrm{sgn}(e_{2})+\hat{\chi }, & \left(e_{2}={\dot{\hat{X}}}_{1}-{\hat{X}}_{2}\right) \end{array}\\ \dot{\hat{\chi }}=\lambda_{0}.\mathrm{sgn}(e_{1})\\ \hat{Y}={\left(K_{\beta }\right)}^{T}{\hat{X}}_{1} \end{array}\right..$$

According to Equations (26) and (45), the estimation error performance of model reference HOSTSMO in REBs can be given as follows:

(46) $$\left\{ \begin{array}{l} {\dot{\tilde{X}}}_{1}={\dot{X}}_{1}-{\dot{\hat{X}}}_{1}\\ {\dot{\tilde{X}}}_{2}={\dot{X}}_{2}-{\dot{\hat{X}}}_{2}\\ {\dot{\tilde{X}}}_{1}={\tilde{X}}_{2}-\lambda_{1}{\left|e_{1}\right|}^{2/3}.\mathrm{sgn}(e_{1})\\ {\dot{\tilde{X}}}_{2}=Χ(X_{1},{\hat{X}}_{2},{\tilde{X}}_{2},u)-\lambda_{2}{\left|e_{2}\right|}^{0.5}.\mathrm{sgn}(e_{2})-\hat{\chi }\\ Χ(X_{1},{\hat{X}}_{2},{\tilde{X}}_{2},u)=\alpha (X_{1},X_{2},u)-\alpha (X_{1},{\hat{X}}_{2},u)+\Delta_{\left(X_{1},X_{2},t\right)}+\delta_{i}+\delta_{o}+\delta_{b}\\ \tilde{Y}={\left(K\right)}^{T}X_{1}-{\left(K_{\beta }\right)}^{T}{\hat{X}}_{1} \end{array}\right..$$

If the system states are bounded as $\left|Χ(X_{1},{\hat{X}}_{2},{\tilde{X}}_{2},F)\right|<{Η}^{+}$, then the sliding gains ($\lambda_{0},\lambda_{1},\lambda_{2}$) can be calculated as follows to guarantee stability and convergence:

(47) $$\left\{ \begin{array}{l} \lambda_{0}=1.1{Η}^{+}\\ \lambda_{1}=1.9\sqrt[{3}]{{Η}^{+}}\\ \lambda_{2}=1.5\sqrt{{Η}^{+}} \end{array}\right..$$

Based on Equation (43) and convergence theory, we have:

(48) $$\begin{array}{l} \Delta_{\left(X_{1},X_{2},t\right)}+\delta_{i}+\delta_{o}+\delta_{b}-\lambda_{2}{\left|e_{2}\right|}^{0.5}.\mathrm{sgn}(e_{2})-\hat{\chi }=0\to \\ \lambda_{2}{\left|e_{2}\right|}^{0.5}.\mathrm{sgn}(e_{2})=0\to \\ \Delta_{\left(X_{1},X_{2},t\right)}+\delta_{i}+\delta_{o}+\delta_{b}>\Gamma \\ if(\delta_{i}=0,\delta_{o}=0,\delta_{b}=0)\to \Delta_{\left(X_{1},X_{2},t\right)}\leq \Gamma \end{array}.$$

Based on (5), in healthy condition $\left(\delta_{i}=0,\delta_{o}=0,\delta_{b}=0\right)$ the residual is defined as follows:

(49) $$\hat{\chi }=\Delta_{\left(X_{1},X_{2},t\right)}\leq \Gamma .$$

Whereas, in faulty conditions, $\delta (t-T_{f})\neq 0$ when $t>T_{f}$, the residual signal is defined by:

(50) $$\hat{\chi }=(\Delta_{\left(X_{1},X_{2},t\right)}+\delta (t-T_{f}))>\Gamma$$

Thus, Γ is defined as the threshold value for normal condition and can be used for fault detection. Therefore, based on the proposed HOSTSMO, the following formulation is used for fault detection in REBs:

(51) $$\left\{ \begin{array}{l} if(\delta_{\left(i,o,b\right)}=0)\to \hat{\chi }\leq \Gamma \\ if((\delta_{\left(i,o,b\right)}\neq 0)\to \hat{\chi }>\Gamma \end{array}\right..$$

**Figure 5:**
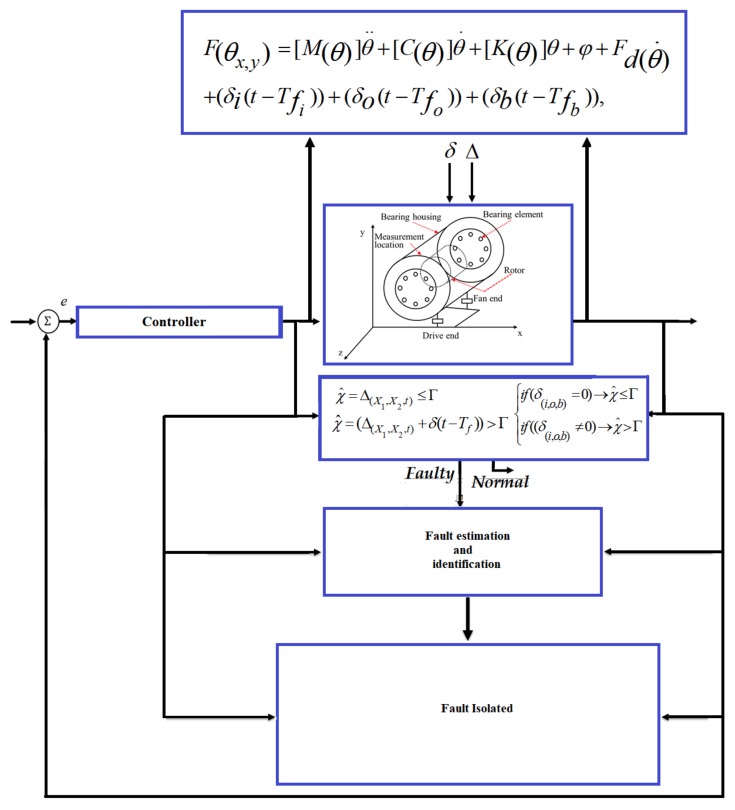
Block diagram of modeling and fault detection of bearing.

**Figure 6:**
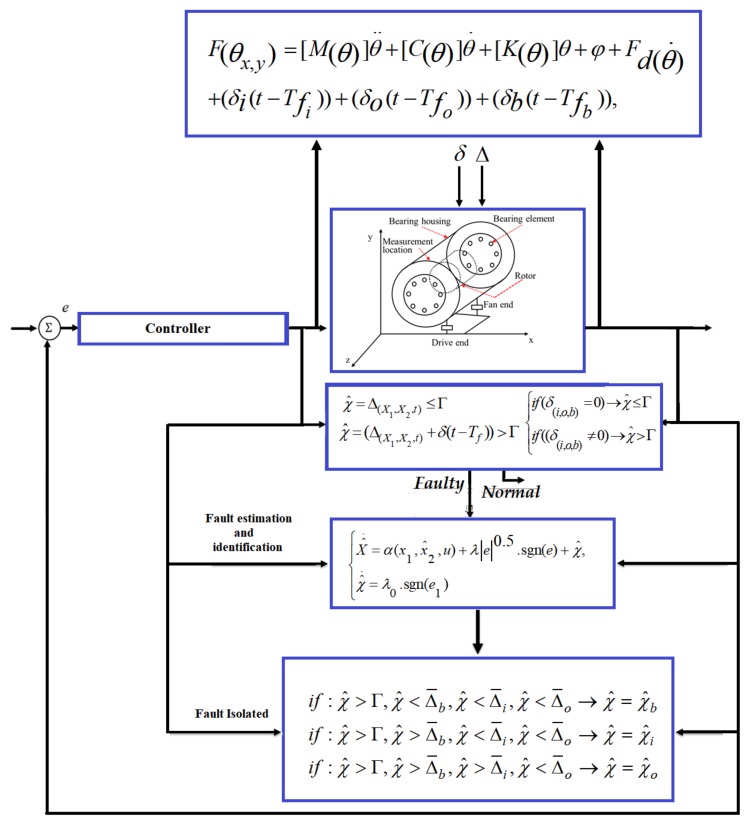
Block diagram of modeling and fault diagnosis of bearing.

The block diagram of the proposed HOSTSMO for fault detection in REBs is given in Figure 5. Based on Equations (26), (45), and (46), the ball, inner, and outer faults are estimated by the proposed HOSTSMO method and defined in (52)–(54), respectively.

(52) $$\left\{ \begin{array}{l} \begin{array}{cc} {\dot{\hat{X}}}_{i_{1}}={\hat{X}}_{i_{2}}+\lambda_{i_{1}}{\left|{e_{i}}_{1}\right|}^{2/3}.\mathrm{sgn}({e_{i}}_{1}), & \left(e_{i_{1}}={X_{i}}_{1}-{\hat{X}}_{i_{1}}\right) \end{array}\\ \begin{array}{l} {{\dot{\hat{X}}}_{i}}_{2}=\alpha_{i}({X_{i}}_{1},{\hat{X}}_{i_{2}},u)+{\lambda_{i}}_{2}{\left|{e_{i}}_{2}\right|}^{0.5}.\mathrm{sgn}({e_{i}}_{2})+{\hat{\chi }}_{i},\\ \left(e_{i_{2}}={\dot{\hat{X}}}_{i_{1}}-{\hat{X}}_{i_{2}}\right) \end{array}\\ {\hat{\dot{\chi }}}_{i}={\lambda_{i}}_{0}.\mathrm{sgn}(e_{i_{1}})\\ {\hat{Y}}_{i}={\left(K_{i}\right)}^{T}{\hat{X}}_{i_{1}} \end{array}\right.,$$

(53) $$\left\{ \begin{array}{l} \begin{array}{cc} {\dot{\hat{X}}}_{o_{1}}={\hat{X}}_{o_{2}}+\lambda_{o_{1}}{\left|{e_{o}}_{1}\right|}^{2/3}.\mathrm{sgn}({e_{o}}_{1}), & \left(e_{o_{1}}=X_{o_{1}}-{\hat{X}}_{o_{1}}\right) \end{array}\\ \begin{array}{l} {\dot{\hat{X}}}_{o_{2}}=\alpha_{o}(X_{o_{1}},{\hat{X}}_{o_{2}},u)+{\lambda_{o}}_{2}{\left|{e_{o}}_{2}\right|}^{0.5}.\mathrm{sgn}({e_{o}}_{2})+{\hat{\chi }}_{o},\\ \left({e_{o}}_{2}={\dot{\hat{X}}}_{o_{1}}-{\hat{X}}_{o_{2}}\right) \end{array}\\ {\hat{\dot{\chi }}}_{o}={\lambda_{o}}_{0}.\mathrm{sgn}(e_{o_{1}})\\ {\hat{Y}}_{o}={\left(K_{o}\right)}^{T}{\hat{X}}_{o_{1}} \end{array}\right.,$$

(54) $$\left\{ \begin{array}{l} \begin{array}{cc} {\dot{\hat{X}}}_{b_{1}}={\hat{X}}_{b_{2}}+\lambda_{b_{1}}{\left|e_{b_{1}}\right|}^{2/3}.\mathrm{sgn}(e_{b_{1}}), & \left(e_{b_{1}}=X_{b_{1}}-{\hat{X}}_{b_{1}}\right) \end{array}\\ \begin{array}{l} {\dot{\hat{X}}}_{b_{2}}=\alpha_{b}(X_{b_{1}},{\hat{X}}_{b_{2}},F)+\lambda_{b_{2}}{\left|e_{b_{2}}\right|}^{0.5}.\mathrm{sgn}(e_{b_{2}})+{\hat{\chi }}_{b},\\ \left(e_{b_{2}}={\dot{\hat{X}}}_{b_{1}}-{\hat{X}}_{b_{2}}\right) \end{array}\\ {\hat{\dot{\chi }}}_{b}=\lambda_{b_{0}}.\mathrm{sgn}(e_{b_{1}})\\ {\hat{Y}}_{b}={\left(K_{b}\right)}^{T}{\hat{X}}_{b_{1}} \end{array}\right.,$$

where $\left({\dot{\hat{X}}}_{i},{\dot{\hat{X}}}_{o},{\dot{\hat{X}}}_{b}),({\hat{\chi }}_{i},{\hat{\chi }}_{o},{\hat{\chi }}_{b}),(\lambda_{i},\lambda_{o},\lambda_{b}\right)$ and $\left(K_{i},K_{o},K_{b}\right)$ are the estimated fault states (inner, outer, ball), (inner, outer, ball) faults and uncertainties estimators, sliding gains for (inner, outer, ball) faults, and output gains for (inner, outer, ball) faults, respectively. Based on (52)–(54), the ball, inner, and outer faults are estimated by the proposed HOSTSMO method. To calculate the threshold values for the ball, inner, and outer fault conditions, a robust partly sliding mode method is used as follows:

(55) $$\begin{array}{l} {\bar{\Delta }}_{b}=-M({\hat{X}}_{1}).K_{\omega_{b}}\mathrm{sgn}(s_{b})\\ s_{b}=\lambda_{b}e_{1_{b}}+{{\dot{e}}_{1}}_{b} \end{array}$$

(56) $$\begin{array}{l} {\bar{\Delta }}_{i}=-M({\hat{X}}_{1}).K_{\omega_{i}}\mathrm{sgn}(s_{i})\\ s_{i}=\lambda_{i}e_{1_{i}}+{{\dot{e}}_{1}}_{i} \end{array}$$

(57) $$\begin{array}{l} {\bar{\Delta }}_{o}=-M({\hat{X}}_{1}).K_{\omega_{o}}\mathrm{sgn}(s_{o})\\ s_{o}=\lambda_{o}e_{1_{o}}+{{\dot{e}}_{1}}_{o} \end{array}$$

where ${\bar{\Delta }}_{b},{\bar{\Delta }}_{i},{\bar{\Delta }}_{o},(K_{\omega_{b}},K_{\omega_{i}},K_{\omega_{o}}),(s_{b},s_{i},s_{o})$ and $\left(\lambda_{b},\lambda_{i},\lambda_{o}\right)$ are threshold values for ball fault, inner fault, outer fault, sliding coefficients for (ball, inner, and outer) faults, sliding surface for (ball, inner, and outer) fault states, and sliding surface slope for (ball, inner, and outer) fault states, respectively. Based on (52)–(57), the ball, inner, and outer faults are identified as follows:

(58) $$\begin{array}{l} if:\hat{\chi }>\Gamma ,\hat{\chi }<{\bar{\Delta }}_{b},\hat{\chi }<{\bar{\Delta }}_{i},\hat{\chi }<{\bar{\Delta }}_{o}\to \hat{\chi }={\hat{\chi }}_{b}\\ if:\hat{\chi }>\Gamma ,\hat{\chi }>{\bar{\Delta }}_{b},\hat{\chi }<{\bar{\Delta }}_{i},\hat{\chi }<{\bar{\Delta }}_{o}\to \hat{\chi }={\hat{\chi }}_{i}\\ if:\hat{\chi }>\Gamma ,\hat{\chi }>{\bar{\Delta }}_{b},\hat{\chi }>{\bar{\Delta }}_{i},\hat{\chi }<{\bar{\Delta }}_{o}\to \hat{\chi }={\hat{\chi }}_{o} \end{array}$$

where ${\hat{\chi }}_{b},{\hat{\chi }}_{i}$ and ${\hat{\chi }}_{o}$ are the residual signals for ball fault, inner race fault, and outer race fault, respectively. Based on (58), the faults are isolated whenever the residuals $\left({\hat{\chi }}_{b},{\hat{\chi }}_{i},{\hat{\chi }}_{o}\right)$ overshoot their corresponding thresholds $\left(\Gamma ,{\bar{\Delta }}_{b},{\bar{\Delta }}_{i}\right)$, respectively. Figure 6 shows the block diagram for fault detection, estimation, identification, and isolation.

## 5. Datasets, Results, and Analysis

**Figure 7:**
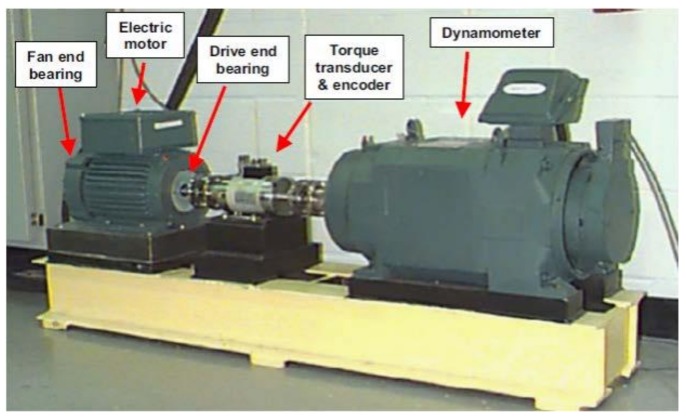
The seeded roller bearing test rig for recording fault data.

To validate the effectiveness of the proposed algorithm, this paper uses the 5-DOF mathematical formulation in [2,52] for REB system modeling and a benchmark bearing dataset that was acquired from Case Western Reserve University (CWRU) [49]. The apparatus employed in the experiment included a 2-hp motor, a torque transducer, a load motor, and a dynamometer. Figure 7 illustrates the detailed location of each component [2]. In this system, the vibration sensor is used for data collection from roller bearings 6205-2RS JEM SKF for the diagnosis of bearing faults. Single-point faults with three different crack sizes (i.e., severity levels) of 0.007, 0.014, and 0.021 inches in diameter were seeded on the drive-end bearings at different bearing locations as the outer raceway fault (OR), inner raceway fault (IR), and the ball fault (Ball), respectively. Data was collected for the three fault conditions and bearings in normal healthy state. The data was recorded at a 12 kHz sampling rate under four different motor loads from 0 to 3 hp. The description of the data is given in Table 2.

**Figure 8:**
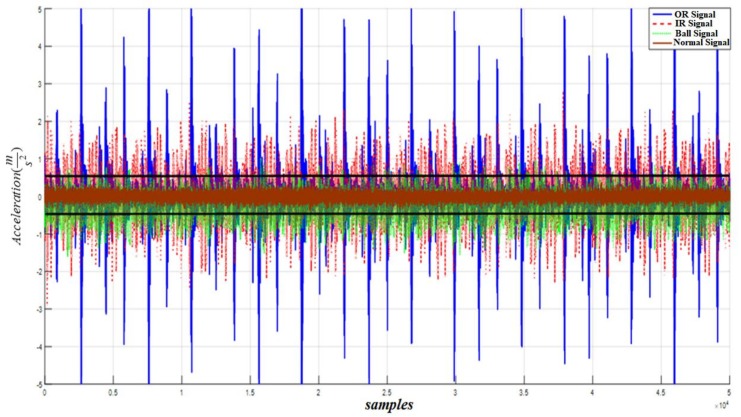
Residual of acceleration for normal, inner, outer, and ball faults and normal threshold value for fault detection.

To validate the efficacy of the proposed HOSTSMO fault diagnosis method, we test it with benchmark bearing datasets as described in Table 2. Figure 8 shows the residual signals for the normal, inner fault, outer fault, and ball fault conditions, and the threshold values for fault detection.

The residual signals are calculated based on the fault models. Based on (55)–(57), the threshold values can be estimated for normal condition (59), ball fault condition (60), inner race fault condition (61), and outer race fault condition (62), respectively.

(59) $$-\Gamma <{\hat{\chi }}_{n}<\Gamma \to -0.5<{\hat{\chi }}_{n}<+0.5$$

(60) $$\{\begin{array}{c} -\Delta_{b}<{\hat{\chi }}_{b}<-\Gamma \\ \Gamma <{\hat{\chi }}_{b}<\Delta_{b} \end{array}\to \left\{ \begin{array}{l} 0.5<{\hat{\chi }}_{b}<1.8\\ -1.8<{\hat{\chi }}_{b}<-0.5 \end{array}\right.$$

(61) $$\{\begin{array}{c} -\Delta_{i}<{\hat{\chi }}_{i}<-\Delta_{b}\\ \Delta_{b}<{\hat{\chi }}_{i}<\Delta_{i} \end{array}\to \left\{ \begin{array}{l} 1.8<{\hat{\chi }}_{i}<3.5\\ -3.5<{\hat{\chi }}_{b}<-1.8 \end{array}\right..$$

(62) $$\{\begin{array}{c} -\Delta_{i}>{\hat{\chi }}_{o}\\ {\hat{\chi }}_{o}>\Delta_{i} \end{array}\to \left\{ \begin{array}{l} {\hat{\chi }}_{o}>3.5\\ {\hat{\chi }}_{o}<-3.5 \end{array}\right.$$

**Figure 9:**
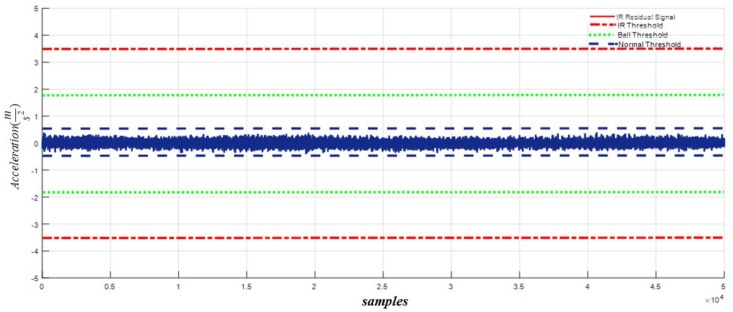
Residual of acceleration for normal signal: normal threshold (±0.5), ball fault threshold (±1.8), and inner fault threshold (±3.5) for a crack width of 0.007.

**Figure 10:**
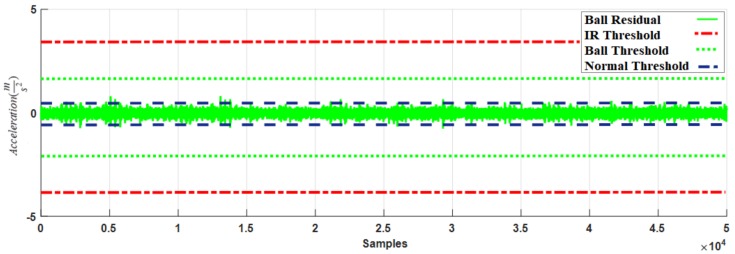
Residual of acceleration for ball fault signal: normal threshold (±0.5), ball fault threshold (±1.8), and inner fault threshold (±3.5) for a crack width of 0.007.

**Figure 11:**
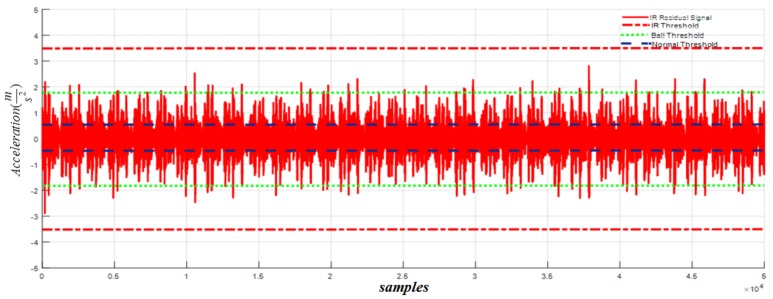
Residual of acceleration for inner fault signal: normal threshold (±0.5), ball fault threshold (±1.8), and inner fault threshold (±3.5) at a crack width of 0.007.

Figure 9 shows the residual signal and threshold values for normal bearings calculated by the proposed HOSTSMO using Dataset 1. The residual signal and thresholds for bearings with a ball fault as calculated by the proposed HOSTSMO technique are displayed in Figure 10. As shown in Figure 10 and given in (60), the ball fault is detected and isolated whenever the residual $\left({\hat{\chi }}_{b}\right)$ overshoots its normal threshold value. The inner fault signal is estimated based on our proposed HOSTSMO estimation technique. The residual signal and threshold range for the inner fault signal are depicted in Figure 11. According to the bearing dynamics, the energy level of the defective inner state is comparatively higher than that of the normal state.

**Figure 12:**
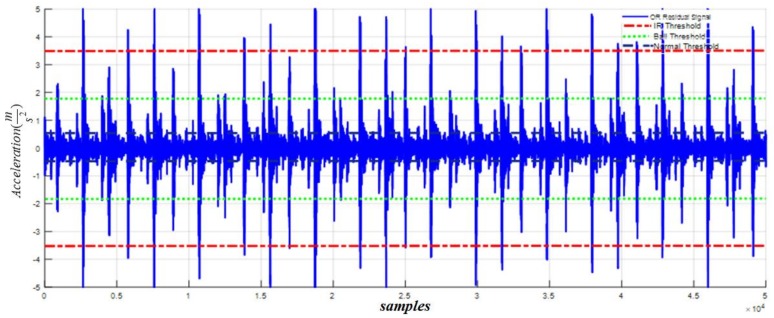
Residual of acceleration for outer fault signal: normal threshold (±0.5), ball fault threshold (±1.8), and inner fault threshold (±3.5) at a crack width of 0.007.

Based on Figure 11 and (61), the inner fault signal is detected and isolated whenever the residual $\left({\hat{\chi }}_{i}\right)$ overshoots the ball threshold value. The outer fault signal for our proposed HOSTSMO estimation technique is illustrated in Figure 12. According to Figure 12 and (62), the outer fault signal is detected and isolated whenever the residual $\left({\hat{\chi }}_{o}\right)$ overshoots the inner threshold value.

According to the results in Figure 8, Figure 9, Figure 10, Figure 11 and Figure 12, we observe that our proposed HOSTSMO technique is highly effective in detecting different fault states. We compare our proposed HOSTSMO method with the state-of-the-art ALPIO technique [57] for performance analysis. To validate our model further, we calculate the diagnostic accuracy for each fault state for the four datasets described in Table 2 under various operating conditions. Table 3, Table 4, Table 5 and Table 6 present the diagnostic performance of the proposed HOSTSMO and ALPIO for each fault type for four datasets. The diagnostic performance is reported as the percentage of correct detections in all data.

As shown in Table 3, Table 4, Table 5 and Table 6, the proposed HOSTSMO-based method for bearing fault diagnosis outperforms the state-of-the-art ALPIO method, yielding average performance improvements of 18.82%, 16.825%, and 17.44% for three fault severity levels characterized by crack sizes of 0.007, 0.014, and 0.021 inches, respectively. This performance improvement can be further validated by the fact that our proposed HOSTSMO model is highly efficient in identifying the signal state and defining the dynamic error threshold as can be seen in Figure 8, Figure 9, Figure 10, Figure 11 and Figure 12.

## 6. Conclusions

This paper presented a nonlinear observation-based bearing fault diagnosis technique using a higher-order super-twisting sliding mode observation method. The bearing fault signal is highly nonlinear and composed of uncertain dynamic parameters, and its vibration measurement is noisy. The filter-less high-order super-twisting sliding mode observation method generates a robust residual signal for the detection, estimation, and identification of the different types of faults found in bearings. To design a robust model-reference observation technique, bearings under normal and faulty conditions were modeled using a 5-degree-of-freedom nonlinear system and applied to the higher-order super-twisting sliding mode observer. The effectiveness of the proposed observation technique was tested with a benchmark dataset that was provided by Case Western Reserve University. The proposed method outperformed the conventional ARX-Laguerre proportional integral observation technique, yielding average performance improvements of 18.82%, 16.825%, and 17.44% for three fault severity levels characterized by crack sizes of 0.007, 0.014, and 0.021 inches, respectively.

## Figures and Tables

**Table 1:** Parameters of REB model.

Parameters	Value
Number of balls	9
Stiffness of ball	5.96 × 10^7^ (Nm)
Mass of outer (Kg)	2.7 (Kg)
Stiffness of outer	1.31 × 10^5^ (Nm)
Mass of shaft (Kg)	1.36 (Kg)
Stiffness of Shaft	23.3 × 10^6^ (Nm)
Damping	654 (NSm)
Ball diameter	7.940 (mm)
Pitch diameter	39.04 (mm)
Defect size	7 (mm)
Defect depth	2 (mm)

**Table 2:** The detailed description of the datasets used in this study.

Dataset	Fault Types	Load (hp)	Fault Crack Sizes (in)
Dataset 1	Normal state	0	0.007, 0.014, and 0.021
	IR fault states	0	
	OR fault states	0	
	Ball fault states	0	
Dataset 2	Normal state	1	0.007, 0.014, and 0.021
	IR fault states	1	
	OR fault states	1	
	Ball fault states	1	
Dataset 3	Normal state	2	0.007, 0.014, and 0.021
	IR fault states	2	
	OR fault states	2	
	Ball fault states	2	
Dataset 4	Normal state	3	0.007, 0.014, and 0.021
	IR fault states	3	
	OR fault states	3	
	Ball fault states	3	

IR = inner raceway fault; OR = outer raceway fault; Ball = ball fault.

**Table 3:** Fault diagnosis results for Dataset 1 for the proposed method and ALPIO when torque load = 0 hp.

Algorithms	Proposed Method				ALPIO	
Crack Diameters (in)	0.007	0.014	0.021	0.007	0.014	0.021
Normal State	100%	100%	100%	89%	89%	89%
IR Faults	96%	93%	96%	66%	70%	70%
OR Fault	100%	100%	100%	75%	80%	78%
Ball Fault	100%	100%	100%	81%	81%	84%
Average	99%	98.3%	99%	78%	80%	80.3%

**Table 4:** Fault diagnosis results for Dataset 2 for the proposed method and ALPIO when torque load = 1 hp.

Algorithms	Proposed Method				ALPIO	
Crack Diameters (in)	0.007	0.014	0.021	0.007	0.014	0.021
Normal State	100%	100%	100%	89%	89%	89%
IR Faults	100%	100%	100%	66%	70%	70%
OR Fault	95%	93%	95%	75%	80%	78%
Ball Fault	98%	90%	98%	81%	81%	84%
Average	98.3%	95.7%	98.3%	78%	80%	80.3%

**Table 5:** Fault diagnosis results for Dataset 3 for the proposed method and ALPIO when torque load = 2 hp.

Algorithms	Proposed Method				ALPIO	
Crack Diameters (in)	0.007	0.014	0.021	0.007	0.014	0.021
Normal State	100%	100%	100%	85%	85%	85%
IR Faults	100%	100%	100%	73%	70%	75%
OR Fault	92%	85%	95%	75%	75%	75%
Ball Fault	93%	90%	90%	78%	81%	81%
Average	96.3%	93.8%	96.3%	77.8%	77.8%	79%

**Table 6:** Fault diagnosis results for Dataset 4 for the proposed method and ALPIO when torque load = 3 hp.

Algorithms	Proposed Method				ALPIO	
Crack Diameters (in)	0.007	0.014	0.021	0.007	0.014	0.021
Normal State	100%	100%	100%	90%	90%	90%
IR Faults	94%	100%	100%	75%	75%	75%
OR Fault	90%	90%	90%	75%	75%	78%
Ball Fault	92%	86%	90%	78%	75%	81%
Average	94%	94%	95%	79.5%	78.75%	81%
